# Deashed Wheat-Straw Biochar as a Potential Superabsorbent for Pesticides

**DOI:** 10.3390/ma16062185

**Published:** 2023-03-09

**Authors:** Irmina Ćwieląg-Piasecka, Elżbieta Jamroz, Agnieszka Medyńska-Juraszek, Magdalena Bednik, Bogna Kosyk, Nora Polláková

**Affiliations:** 1 Institute of Soil Science, Plant Nutrition and Environmental Protection, Wroclaw University of Environmental and Life Sciences, Grunwaldzka 53 St., 50-357 Wrocław, Poland; 2 Faculty of Agrobiology and Food Resources, Institute of Agronomic Sciences, Slovak University of Agriculture, Tr. A. Hlinku 2, 949 76 Nitra, Slovakia

**Keywords:** biochar modification, deashing, pesticides, 2,4-D, MCPA, carbaryl, carbofuran, metolachlor, sorption

## Abstract

Biochar activation methods have attracted extensive attention due to their great role in improving sorptive properties of carbon-based materials. As a result, chemically modified biochars gained application potential in the purification of soil and water from xenobiotics. This paper describes changes in selected physicochemical properties of high-temperature wheat-straw biochar (BC) upon its deashing. On the pristine and chemically activated biochar (BCd) retention of five pesticides of endocrine disrupting activity (carbaryl, carbofuran, 2,4-D, MCPA and metolachlor) was studied. Deashing resulted in increased sorbent aromaticity and abundance in surface hydroxyl groups. BCd exhibited more developed meso- and microporosity and nearly triple the surface area of BC. Hydrophobic pesticides (metolachlor and carbamates) displayed comparably high (88–98%) and irreversible adsorption on both BCs, due to the pore filling, whereas the hydrophilic and ionic phenoxyacetic acids were weakly and reversibly sorbed on BC (7.3 and 39% of 2,4-D and MCPA dose introduced). Their removal from solution and hence retention on the deashed biochar was nearly total, due to the increased sorbent surface area and interactions of the agrochemicals with unclogged OH groups. The modified biochar has the potential to serve as a superabsorbent, immobilizing organic pollutant of diverse hydrophobicity from water and soil solution.

## 1. Introduction

In recent years, biochar has been widely investigated as a nutrient carrier with slow-release capacity, an additive reducing soil water loss, or sorbent of various xenobiotics [1,2,3]. The most important properties of biochar determining its high adsorptive potential are: large surface area, porous structure, abundance in surface functional groups, and high cation exchange capacity [4]. These features may induce chemical immobilization of contaminant molecules on biochar and as a result mitigate their leaching and bioavailability. Hence, a vast majority of biochar studies have been devoted to contaminated water treatment and soil remediation [5,6]. Many attempts have also been made to modulate the basic pyrolysis process parameters (input material, temperature, time) to obtain modified biochar [7,8]. The aim of that was to design a material with properties allowing for the retention of particular groups of pollutants, such as heavy metals [9], polycyclic aromatic hydrocarbons [10], pesticides [11], pharmaceuticals, or personal care products [12]. In subsequent studies, many chemical and physical activation methods of biomaterials (both on the pre-pyrolyzed biomass or pristine biochar produced) have been implemented to produce the biosorbent of the desired properties [13,14,15].

Among the chemical activation methods of biochar, deashing has recently been gaining more attention in terms of retention of hydrophobic contaminants on high temperature biochars. Generally, ash content in biochar obtained from plant residue is lower (up to 20–30%) than in animal-derived biosorbents (up to even 90%) [16,17,18], and its share increases significantly with pyrolysis temperature [19]. This inorganic BC fraction comprises high concentrations of various mineral constituents such as Ca, K, Mg, Si and Na, etc. in the form of oxides, phosphates, carbonates and silicates [18]. Such properties facilitate its use in agriculture as a liming agent and an amendment abundant in some macro- and micronutrients, especially in the case of sludge and animal-derived biochars [20,21,22,23]. In contrast, high-temperature (>500 °C) plant-derived biochars are more commonly used to mitigate soil contamination via immobilization of heavy metals and organic pollutants [24,25,26]. In this case, a high level of ash in the sorbent biomass can obstruct pores of biochar and consequently decrease its surface area and sorption capacity [27]. Zhang et al. [28] found that ash in biochars can bind neonicotinoids by specific interactions, but had an adverse effect on sorption magnitude, due to the covering of the inner sorption sites of organic moieties and blocking the micropores. Deashing may significantly increase core carbon moieties, bulk oxygen, aromaticity and O-containing functional groups, decreasing all surface element contents at the same time [28]. Removal of inorganic compounds, which block some organic adsorption sites in the pristine biochar, has been proven a successful strategy to increase sorption of several organic compounds in soils e.g., phenantrene [29], naphthalene or 1-naphthol [30], pyrazysulfuron-ethyl herbicide [31], neonicotinoid insecticides [28], bispyribac sodium and clomazone herbicides [32]. Nevertheless, there has been no universal sorbent designed so far that could be dedicated to a wide spectrum of chemical classes of pollutants.

Essentially, biochar can sorb organic molecules in two ways based on sorbate–sorbent interaction strength. Usually, at the beginning, partitioning or pore filling is performed jointly with other physisorption interactions such as hydrophobic interactions, π–π bonds, van der Waals forces or hydrogen bonding. These mechanisms are mostly reversible, weak intermolecular physical interactions [33]. At the consecutive stage, chemisorption may play a role through covalent bonding or complex formation, which are irreversible monolayer chemical interactions [11]. Therefore, pesticide sorption on biochar matrix is often the result of synergistic mechanisms, depending on physicochemical attributes of the sorbent and agrochemical properties.

Endocrine disrupting compounds (EDCs) are defined as chemicals that can mimic endogenous hormones or interfere with endocrine processes [34]. Adverse effects of EDCs (particularly xenoestrogens) include a number of developmental anomalies in wildlife and humans. They belong to the group of contaminants of emerging concern (CECs) that encompass a broad category of pollutants, including also pharmaceuticals and personal care products (PPCPs), flame retardants (FRs), pesticides, and artificial sweeteners (ASWs) [35]. They have all been detected in aquatic environments and may cause ecological or human health impacts. Pesticides chosen for this study—2,4-D, MCPA, carbofuran, carbaryl and metolachlor—can all be classified as endocrine disruptors [36,37,38,39], and soil is an important environmental sink for these substances [40,41,42,43]. They have distinct physiochemical properties, which is why finding a relatively recalcitrant biosorbent (as a filtering material or soil amendment) that will effectively immobilize both polar and hydrophobic organic xenobiotics is a challenge.

The aim of this study was to evaluate the process of chemical activation of a wheat-straw biochar on selected properties of the biosorbent. Subsequently, retention of investigated endocrine-active pesticides on the pristine and modified biochars was estimated. Changes in sorbent surface properties with deashing were correlated with its potential to immobilize studied agrochemicals of various hydrophobicity. 

## 2. Materials and Methods

### 2.1. Chemicals Used in the Studies

Five pesticides, belonging to three chemical classes, of various hydrophobicity and water solubility were chosen for the studies: nonionic carbamates (carbaryl (1-naphthyl-N-methylcarbamate, 97%) and carbofuran (2,3-dihydro-2,2-dimethyl-7-benzofuranol N-methylcarbamate, 98%)) and aniline derivatives (metolachlor solution in acetonitrile 100 μg/mL and (2-chloro-N-(2-ethyl-6-methylphenyl)-N-(−2-methoxy-1-methylethyl)acetamide, 98%), as well as ionic herbicides belonging to the phenoxyacetic acids (2,4-dichlorophenoxyacetic acid (2,4-D, 98%) and 4-chloro-2-methylphenoxyacetic acid (MCPA, 99%)). The DPPH (2,2-diphenyl-1-picrylhydrazyl) radical, Folin–Ciocâlteu reagent, gallic acid, and active substances of agrochemicals were of analytical grade and purchased from Merck (Schnelldorf, Germany). Their basic chemical and physical properties can be found in our previous study [24]. Additionally, CaCl_2_, NaOH, HCl and 96% ethanol (all of analytical purity) were supplied by Avantor Performance Materials (Gliwice, Poland). Ultrapure water, used to prepare all the solutions, was generated in a Merck Millipore Direct Q3 system (Warsaw, Poland).

### 2.2. Deashing Procedure of a Wheat Straw Biochar

The biochar used in the research was produced from wheat straw, which was subjected to pyrolysis at 550 °C for 30 s in conditions of limited oxygen availability. The material was then ground and passed through a sieve with a mesh diameter of 2 mm, thus obtaining a fine powder consistency. The material was kept in a glass bottle, marked BC, and stored at room temperature until use.

In the next step, the biochar was chemically activated in the ash removal process according to the procedure described by Sun et al. [29] with some modifications. The deashing of biochar was based on its demineralization with 1 M HCL and 10% (*v*/*v*) HF at a ratio of 1:20 (solid/liquid). Then, the mixture was shaken at 25 °C and 140 rpm for 5 days. After that, the supernatant (obtained after centrifugation of the tested mixture at 4500 rpm for 30 min) was removed. The entire procedure was repeated six times to obtain deashed BC samples with a sufficiently low ash content, which was monitored throughout the experiment. Then, the samples were neutralized with demineralized water and freeze-dried to obtain a powdered, deashed biochar marked as BCd.

### 2.3. Properties of the Studied Sorbents

Selected basic properties of pristine (BC) and deashed (BCd) biochars were examined according to the methodology described below. They were then compared to observe the changes in particular properties of the sorbents caused by the biochar demineralization process.

The pH was measured potentiometrically in 10 mM CaCl_2_ at a ratio of 1:20 (*m*/*v*, 24 h with stirring) [44]. The elemental composition was determined on a CE EA 1110 CHNS instrument. Oxygen content was calculated from the weight difference (O% = 100 − (CHNS + ash)). Based on these results, molar ratios of H/C and O/C were calculated (Table 1). By burning the sorbent samples at 550 °C for 6 h [45], their ash content was estimated. All measurements were performed in triplicate.

The infrared spectra (FTIR) of the pristine and modified biochar samples were recorded on KBr pellets using a Vertex 70 FT-IR spectrometer (Bruker, Billerica, MA, USA) over the wavenumber range 4000–400 cm^−1^.

The specific surface area and the parameters of the porous structure were determined using the nitrogen sorption method in 77 K (N_2_-BET method). Measurements were made on an Autosorb IQ apparatus (Quantachrome, Boynton Beach, FL, USA). The calculations included S_BET_—the specific surface area determined from the Brunauer–Emmett–Teller equation [m^2^ g^−1^], V_T_—total pore volume determined using Gurvitch’s law from the amount of adsorbed gas at relative pressure p/p_0_ = 0.96 [cm^3^ g^−1^], V_micro_—volume of micropores calculated from the Dubinin–Raduszkiewicz equation [cm^3^ g^−1^] and V_meso_—mesopore volume calculated from the V_T_-V_micro_ difference [cm^3^ g^−1^] [46].

The surface morphology of the studied biochars was investigated using scanning electron microscopy (SEM; EVO LS 15, Carl Zeiss) and EHT of 20 kV. Energy-dispersive X-ray (EDX) spectroscopy was used to determine the elemental composition of both biochars. Elemental analyses and the distributions of elements were performed at magnifications of 1000× using a Bruker Quantax 200 system with a Bruker X Flash detector 5010.

Spectrophotometric UV-vis methods were applied to study the antioxidant potential of the tested biosorbents using DPPH radical scavenging and Folin–Ciocâlteu assay [47,48]. Analyses were conducted on BC and BCd extracts. In the first step, biochars were subjected to a 6 h microwave-assisted extraction procedure in 0.1 M NaOH and ethanol at 40 °C. Then, the alkaline extracts of BC and BCd were neutralized with 1 M HCl solution and—in parallel with alcoholic extracts—filtered through 0.45 µm membrane filters.

The DPPH free radical scavenging activity of pristine and deashed biochar extracts (NaOH-neutralized and ethanolic) was estimated by mixing aliquots of 1.5 mL of obtained extracts of BC and BCd samples with 1.5 mL ethanol solution of 0.1 mM DPPH. The mixture was allowed to stand for 30 min in the dark. The absorbance was measured at 523 nm using a UV-vis spectrophotometer (Cary 60, Varian, Wroclaw, Poland). An equal amount of DPPH and ethanol were used as a control and blank, respectively. The measurements were done in triplicate. The scavenging activity was calculated using the following equation [49]:

Scavenging (%) = (Ac − As)/Ac·100(1)

where As is the absorbance of the tested extracts and Ac is the absorbance of the DPPH control solution.

Folin–Ciocâlteu assay was performed for both types of extracts of BC and BCd. Triplicate samples of 1 mL of neutralized alkaline and ethanolic extracts, both diluted with 4 mL of deionized water, were mixed with 1 mL of FC reagent and—after 3 min—with 3 mL of 20% Na_2_CO_3_. They were left to stand in the dark for 60 min, followed by measurement of the absorbance at 765 nm (Cary 60, Varian, Wroclaw, Poland). To quantify the measurements, a calibration curve was prepared, where total phenolic content was expressed as gallic acid equivalent (mg of GA L^−1^ of extract; Supporting Materials, Figure S1).

### 2.4. Sorption–Desorption Experiment

Sorption of the investigated pesticides on the wheat-straw biochar and its deashed counterpart was determined using a simplified batch equilibrium method [24,50]. To compare the effectiveness of the two sorbents in selected agrochemical retention, a single concentration of the pesticides was applied (approx. 10 mg L^−1^ for carbofuran, metolachlor and MCPA and 12.5 mg L^−1^ and 17.1 mg L^−1^ for 2,4-D and carbaryl, respectively).

In the sorption experiment, BC and BCd biochars (50 mg aliquots) were shaken with 10 mL of pesticide solutions for 24 h, and then centrifuged at 10,000 rpm. The supernatant was then taken up, additionally passed through a 0.45 µm membrane filter and analyzed for the particular pesticide content using liquid chromatography coupled with tandem mass spectrometry (LC-MS/MS). Conditions of the measurements are given elsewhere [24]. At this stage, the pH of the supernatant solution after 24 h of sorption was measured in both variants with BC and BCd as sorbents. After removal of the supernatant, samples were treated with an equivalent amount of 10 mM CaCl_2_, shaken for another 24 h, centrifuged, and the solution with the desorbed amount of pesticide subjected to LC-MS/MS measurements. The desorption was defined as the percentage of the test substance released back to the solution, related to the quantity of substance previously adsorbed, under the test conditions [50]. One desorption step was performed for each sample. Additionally, control samples containing pesticide solutions without the tested sorbents, with pH adjusted to that experimentally measured, were prepared. This was done to exclude chemical hydrolysis as a competitive mechanism to adsorption. All the measurements were done in triplicate.

The amounts of pesticides adsorbed by each biochar were determined from the difference between the initial and final concentrations of the sorbates in the solutions, using the following equation [24,51]:

Q = [(C_0_ − C_eq_)·V]·m^−1^ [mg g^−1^] (2)

where Q is the adsorption capacity, expressed as mg of pesticide per g of biochar, C_0_ is the initial pesticide concentration (mg L^−1^), C_eq_ is the pesticide concentration at equilibrium, measured after 24 h of contact with the adsorbent (mg L^−1^), V is the volume of the solution used in the experiment (L), and m is the weight of biochar used (g).

The sorption magnitude of each of the particular agrochemical is equivalent to percentage of removal of the pesticides from the investigated solutions. It was calculated as the difference between C_0_ (initial concentration of the pesticide) and C_eq_ (concentration after the contact with the adsorbent) related to C_0_. Sorption magnitude was expressed in percentages and estimated with the following equation:

Sorption = [(C_0_ − C_eq_)·C_0_^−1^]·100 [%] (3)

### 2.5. Statistical Analysis

Data are expressed as the mean values of three replicates ± standard deviation (SD). Dixon’s Q-test [52,53] was applied to identify potential outliers among the obtained results at a confidence level of 95%. Mean values of the pesticides’ sorption and desorption magnitude on pristine and deashed biochars, adsorption capacity, as well as DPPH and Folin–Ciocâlteu assay results, were statistically compared using the Student’s *t*-test (at *p* < 0.05). Data were compiled using Microsoft Excel 2019 and GraphPad Prism 9.5.1.733 software.

## 3. Results and Discussion

### 3.1. Basic Material Properties

The basic properties of a wheat-straw biochar before (BC) and after demineralization in HF (BCd) are presented in Table 1.

The pristine biochar (BC) was highly alkaline, and its pH value was 8.97. It was influenced by the separation of alkali salts from the organic matrix in the input material during the pyrolysis process. The ash of the high-temperature biochars consists mostly of CaCO_3_, NaCl, and KCl as well as silicates [54]. Its relatively high share (28.08% m/d.m.) in BC can be attributed to the initial inorganic component content in the input material, as well as combustion of cellulose and hemicellulose, which in wheat straw constitute up to 34–40% and 20–25% of a dry mass, respectively [55]. After the HF/HCl treatment, the pH value of biochar dropped significantly to 2.16 (BCd), which confirms the removal of alkaline elements from the pristine BC. The most pronounced demineralization effect was a decrease in ash content by nearly 24% to 4.3% (m/d.m., Table 1). This corroborates the utility of the method in removing inorganic constituents. Moreover, the share of carbon with deashing significantly increased, from 59.72% to 78.18%, as well as the hydrogen (from 1.78% to 1.96% m/d.m.) and oxygen content (from 9.81% to 14.68% m/d.m.). This was the effect of BCd mass contraction (by approx. 24%) with mineral removal.

H/C and O/C molar ratios of both biosorbents studied were smaller than 0.7 and 0.4, respectively, which is in agreement with the IBI (International Biochar Initiative) definition of biochar [56]. The IBI is a civil society organization established in 2007 in the US that promotes systems to produce and utilize biochar as well as coproduction of clean energy (https://www.unccd.int/cso/international-biochar-initiative, accessed on 1 March 2023). Similarly, EBC (European Biochar Certificate, https://www.carbon-standards.com/en/home, accessed on 1 March 2023) guidelines for sustainable biochar production [45] assume that 0.7 is the maximum value of H/C ratio for BC. According to them, values exceeding 0.7 are an indication of non-pyrolytic chars or pyrolysis deficiencies. Generally, it is presumed that biochars that are formed above 400 °C should have an H/C ratio lower than 0.5. Its drop below 0.3 (after demineralization) suggests a relative increase in material aromaticity and hence a greater share of the fused aromatic ring structures [24,57]. The molar O/C ratio of the BCd slightly increased, which indicates the higher polarity of the material in comparison to pristine biochar (BC) [58]. The observed trend of the (O/C) ratio suggests that the surface of the studied BCd biochar may became more hydrophilic with deashing. What is more, according to Spokas et al. [59], the O/C ratio is not only a function of production temperature but also accounts for other impacts such as parent material and postproduction oxidation. This in turn determines material stability and reflects its carbon sequestration potential. Thus, biochars with O/C ratios < 0.2 are believed to be highly stable with half-life exceeding 1000 years [60], which was the case for both tested sorbents.

### 3.2. FTIR Analysis

**Figure 1:**
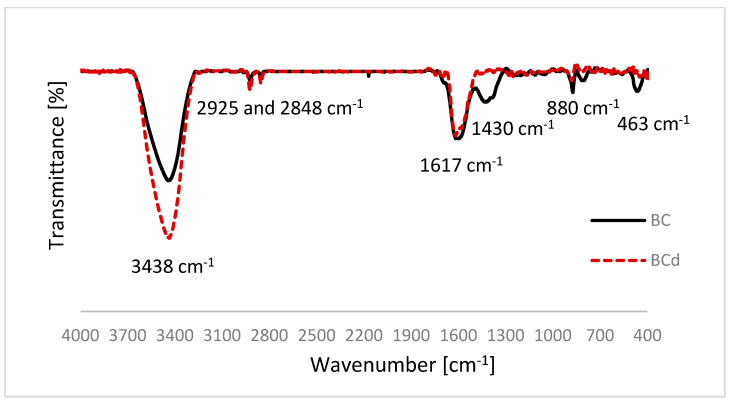
Infrared absorption spectra of the BC (solid line) and BCd (dashed line) biochars.

The FTIR spectra of the biochars (Figure 1) showed a broad band at 3200–3400 cm^−1^, attributed to the O–H stretching vibration of hydroxyl functional phenolic groups, including hydrogen bonding due to adsorbed water. Increase in relative intensity in the BCd sample may be related to uncovering of the surface functional OH groups due to the removal of the mineral components from BC, which facilitates the chemical binding of ionic molecules. For both biochars, absorption in the region between 2925 and 2848 cm^−1^ was assigned to C–H stretching vibration from the methyl and methylene groups [61]. An intense band at 1617 cm^−1^ is related to the vibrations of aromatic C=C and C=O groups of conjugated ketones and quinones [31]. Bands at 1430 and 1377 cm^−1^ can be assigned to aliphatic C-H folding. Its depletion in BCd sample may be the result of removal of original organic matter, such as polymeric CH_2_ and fatty acid, lignin and some cellulose with demineralization, which was also supported by the decrease in the H/C ratio [62]. The weak bands between 885 and 750 cm^−1^ can be attributed to =CH out-of-plane vibrations of the aromatic ring. The Si-O-Si bending vibrations present in BC sample at 463 cm^−1^ diminish after demineralization (BCd) due to the nearly total removal of silicates (Supplementary Materials, Table S1). According to the literature, these vibrations, providing the information mainly on the quartz part of the inorganic matrix, tend to appear in the range of ~470–430 cm^−1^ [63,64].

### 3.3. Porosity and Surface Area Studies

Demineralization resulted in an increase in total surface area of wheat-straw biochar by a factor of 2.7 (Table 2), achieving a final value of 250 m^2^ g^−1^ in BCd. The total pore volume (V_T_) parameter became triple that of before the chemical modification of the biosorbent, reaching 0.24 cm^3^ g^−1^. This was due to the removal of mineral components from the biomass surface, which unclogged pores in the resulting biochar (BCd) structure. Therefore, the access to available active sites remarkably increased. An interesting example of biochar modification also leading to the increase of the surface area of the sorbent was the work of Qu et al. (2022, [9]). Therein, functional PSB bacteria were immobilized on chemically modified bone char, which generated more active sites on its surface. Increased pore volume along with the higher surface area of the BCd might induce good mechanical interlocking between the solute and the biosorbent matrix [62]. However, when discussing the sorption mechanism of organic molecules, the type and relative share of the pore types, particularly the meso- and micropores, is also significant. Recently, a modified β-cyclodextrin sorbent with greatly enhanced sorptive properties due to the formation of microspheres on the sorbent surface was designed (2023, [65]). In the modified biosorbent (BCd), the content of the micropores and mesopores with deashing increased 2.5 and 3.5 times, respectively. The sorbent became slightly more mesoporous as their percentage share increased from 50% to 58.3% (Table 2). The deashed BC, due to the high total surface area and abundance of micro- and mesopores, gained the potential to immobilize tested chemicals via the physical diffusion mechanism, as they may be physically entrapped within the pores on its surface.

### 3.4. SEM Analysis Results

**Figure 2:**
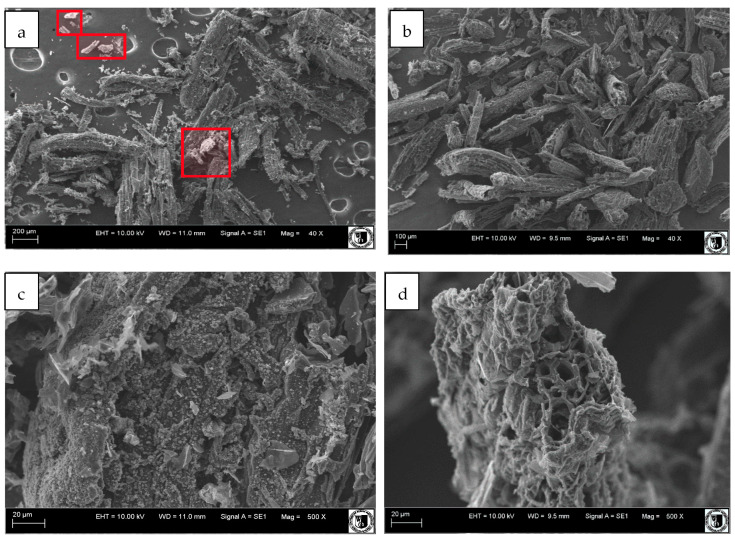
Scanning electron microscopy images amplified 40 times (**a**,**b**) and 500 times (**c**,**d**) of the pristine (**a**,**c**) and deashed biochar (**b**,**d**). Ash particles are highlighted in red.

SEM images of the prepared biochar samples are presented in Figure 2. The SEM images show that BC samples (Figure 2a,c) have randomly shaped structures with sharp-edged particles (ash particles highlighted in red). It can be observed that the HF/HCl treatment did not significantly change the particle size of biochar, but visibly reduced the ash content (Figure 2a,b). Additionally, high-magnification images (Figure 2c,d) clearly display the more porous structure in the deashed biochar (Figure 2d). Here also, the appearance of unclogged pores became more prominent. The porous structure in biochar is of vital importance in terms of effective mechanical interlocking and formation of stronger BC–organic molecule interfaces [62,66].

Energy-dispersive X-ray (EDX) spectroscopy, used to determine the elemental composition of both biochars, confirmed the removal of silicates and decrease in elemental oxygen (Supporting Materials, Table S1). As has been reported in the literature, in high-temperature biochars, besides O-containing mineral compounds (originating from the source biomass), a particular share of the oxygen atoms is of existing functional groups [27]. As the mineral constituents exhibit high susceptibility to HCl/HF treatment, which corroborates the significant decline in BC’s inorganic fraction, the remaining elemental oxygen share in BCd can be attributed mostly to the O-containing organic functional groups. This is in accordance with the FTIR results (Figure 1), which confirmed the relative increase in abundance of -OH groups and absence of silicates in the BCd sample.

### 3.5. Antioxidant Properties of the Biosorbents

The DPPH radical is commonly used to determine the antioxidant activity of different extracts of plant origin [67,68]. It was used in this study due to its advantage over the other analytical methods: it reacts slowly with the whole sample, even with weak antioxidants, and can be used to examine both hydrophilic and lipophilic antioxidants [47]. To the best of our knowledge, it has never been used in assessing biochar’s antioxidant properties. Alkaline extracts of the investigated biochars (after neutralization) exhibited higher antioxidant activities than their ethanolic counterparts (Table 3). General extracts obtained from deashed biochar revealed smaller antioxidant potential (2.78% and 7.53% for ethanolic and NaOH extracts, respectively) than the biosorbent before demineralization. This might be due to the higher content of residual organic matter and inorganic components in pristine biochar capable of donating H atoms and thus reducing the DPPH radical concentration. As such, biochar deashing slightly decreased the initial, already limited antioxidant activity of the investigated pristine wheat-straw biochar. What should be emphasized is that the scavenging activity of both biochar samples is scarce in comparison to those found for plant extracts [69,70].

Folin–Ciocâlteu assay supplementary to DPPH test confirmed low antioxidant potential of both investigated biochars. Usually, the method is utilized to assess the total phenolic content (TPC) of the extract and its antioxidant properties [71,72], expressed in equivalents of the standard substances. Although pristine and deashed biochar extracts gave quantitively measurable results, estimated TPC content did not exceed 5 µg mL^−1^ of the gallic acid equivalents (Table 3). This may suggest that after deashing, the biochar contained fewer compounds of reductive ability capable of reacting with the Folin–Ciocâlteu reagent.

### 3.6. Results of the Sorption Experiment

Structural parameters related to porosity and ash content of both biochars as well as hydrophobicity of pesticides were found to significantly influence the adsorption efficiency. In the sorption experiment, the measured pH of the equilibrated mixtures was maintained below 7 (6.98 and 3.28 for BC and BCd in 10 mM CaCl_2_, respectively). Such conditions prevented carbamate hydrolysis and ensured that the pesticide concentration decline was due to their sorption on biochar. Before the ash removal, biochar was characterized by a particularly low sorption of ionic pesticides of 7.3% and 39.3% for 2,4-D and MCPA, respectively (Table 4). At the same time, their desorption magnitude (expressed as per the amount of pesticide initially sorbed) exceeded 75%, which according to the OECD guideline for testing of chemicals (Adsorption–Desorption Using a Batch Equilibrium Method, [50]) suggests that the adsorption should be considered reversible. After demineralization, removal potential of biochar increased significantly—to 94.4% and 97.5% of the initial 2,4-D and MCPA dose, respectively (Table 4). Particularly pronounced advancement in phenoxyacetic acid retention by BCd becomes evident when their adsorption capacities (Q) are compared (Table 5). Deashing increased the amount of 2,4-D sorbed by a factor of 14.5 and MCPA by 2.5 per 1 g of sorbent. At the same time, desorption of the agrochemicals was significantly reduced (5–13.2%), which indicates that the forces holding the retained pesticide molecules to the solid surface were relatively strong.

In general, pore filling induced by the large surface area of the biochar is postulated as the dominant adsorption mechanism for carbonaceous materials of H/C values below 0.5 [73]. Previously discussed changes in the atomic ratios of H/C and O/C resulting from the demineralization of the BC sample indicate an increase in biochar aromaticity, and at the same time may suggest a slight increase in the share of chemisorption in the binding of the tested phenoxyacetic acids in relation to physical sorption (van der Waals interactions, London forces) [11]. Increased SSA of BCd and pore unclogging may have compensated previously reduced ability of surface OH functional groups to form chemical bonds. This is in accordance with the FTIR results (Figure 1), where the enhanced abundance of OH band was observed after biochar deashing. These joint changes may explain the increase in the chemisorption potential of BCd in relation to the ionic and polar compounds studied.

The investigated carbofuran and metolachlor were characterized by the slightly increased degree of adsorption on biochar after the demineralization process (92.7% and 96.4%, respectively). Nevertheless, the differences in their sorption magnitude on pristine and deashed biochar were not statistically significant (Table 4), hence the HF/HCl treatment did not influence initially high biochar affinity for these substances. However, in carbaryl, a small and significant decline in pesticide sorption with biochar deashing can be observed. In general, investigated carbamate desorption from deashed BC reached slightly higher values (3.1% and 5.9% for carbaryl and carbofuran, respectively) than on pristine sorbent. Increased BCd polarity may be the reason for the weakening of the forces holding this group of agrochemicals. Due to metolachlor and carbamates being more hydrophobic with fewer polar characteristics than phenoxyacetic acids, it is presumed that they were preferentially bound by both sorbents via combined physisorption mechanisms such as pore-filling and hydrophobic interactions. In metolachlor, the additional H-bonding between the agrochemical and unclogged oxygen-containing functional groups present on the surface of biochar may have occurred. Adsorption capacities of the carbamates and metolachlor (Table 5) did not exhibit a relatively high increase or decline after sorbent deashing, and thus the high biochar removal ability towards these pesticides was maintained.

**Figure 3:**
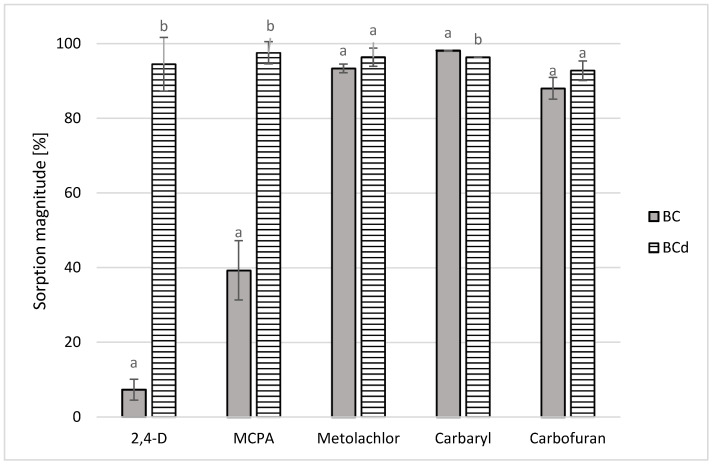
Comparison of the sorption magnitude of the tested pesticides on biochars before (BC) and after ash removal (BCd). Error bars represent ± values of the standard deviations of triplicate samples. Letters a and b indicate significant differences between sorption magnitude of each pesticide on pristine and deashed biochar (*p* < 0.05).

For a better visualization of the results, they are also summarized in Figure 3, which shows a comparison of the sorption magnitude of the tested pesticides on biochars before (bars on the graph marked as BC) and after ash removal (bars marked with BCd).

Comparing both sorbents (BC and BCd) in terms of the type of groups of organic compounds effectively retained by them, it can be noticed that the pristine biochar preferably sorbs the hydrophobic nonionic pesticides (carbaryl, carbofuran, metolachlor). Its modified counterpart became superabsorbent, irreversibly retaining all three classes of organic compounds to the extent of over 90%.

Improved physicochemical properties of the activated wheat-straw biochar make it a promiscuous sorbent for hydrophobic and hydrophilic xenobiotics in water and soil environments. Nevertheless, further studies performed on the BCd-amended soils should be conducted to verify its applicable character. The presented results may serve as preliminary guidance for selecting suitable biochars (pristine or deashed) according to their applications in the environment.

## 4. Conclusions

Wheat-straw biochar deashing is an effective method of its chemical activation, resulting in a higher degree of BCd carbonization in comparison to BC. Ash removal caused an increase in biochar specific surface area due to unclogging of the inner pores. The resulting sorbent was more aromatic, rich in micro- and mesopores, and more abundant in surface hydroxyl groups than the pristine one. BCd revealed weaker antioxidant properties than BC, as with deashing the residual organic matter was removed.

Due to the unclogging of the inner pores with HF/HCl treatment, the potential sorption sites were more available for the tested agrochemicals. As a result, the previously obstructed functional groups of biochar were activated and able to chemically bind the investigated phenoxyacetic acids. We postulate that after deashing, this form of sorption had a significant share of ionic polar pesticide retention jointly with physical sorption mechanisms. Hydrophobic interactions and pore filling were crucial in carbamates and metolachlor retention on both biochars. The conducted studies allowed us to select BCd as the most effective among the tested sorbents for removing both ionic and nonionic pesticides of different water solubility and octanol–water partition coefficient.

## Figures and Tables

**Table 1:** Basic properties of pristine wheat-straw biochar (BC) and its deashed counterpart (BCd). Shares of the C, H, N, S and O elements are expressed in % weight per dry mass weight.

Sample	pH (CaCl_2_)	Ash Content (% m/dm)	C (% m/dm)	H (% m/dm)	N (% m/dm)	S (% m/dm)	O (% m/dm)	H/C (Molar Ratio)	O/C (Molar Ratio)
BC	8.97	28.08	59.72	1.78	0.31	0.30	9.81	0.355	0.123
BCd	2.16	4.30	78.18	1.96	0.71	0.17	14.68	0.298	0.141

**Table 2:** The specific surface area and the porous structure characteristics of BC and BCd samples.

Sample	S_BET_	V_T_	V_micro_	V_meso_	Micro ^a^	Meso ^a^
	[m^2^ g^−1^]	[cm^3^ g^−1^]	[cm^3^ g^−1^]	[cm^3^ g^−1^]	Porosity (%)	Porosity (%)
BC	92	0.08	0.04	0.04	50.0	50.0
BCd	250	0.24	0.10	0.14	41.7	58.3

S_BET_—the specific surface area m^2^ g^−1^; V_T_—total pore volume; V_micro_—volume of micropores, V_meso_—mesopore volume. ^a^ Microporosity (%) = (V_micro_/V_T_) 100%; ^a^ mesoporosity (%) = (V_meso_/V_T_) 100%.

**Table 3:** Calculated scavenging activity of pristine and deashed biochar extracts (ethanolic and alkaline after neutralization). Results are expressed as mean values ± standard deviation (*n* = 3).

Test Performed	BC_EtOH_	BCd_EtOH_	BC_NaOH_	BCd_NaOH_
Scavenging activity [%]	5.57 ± 0.15 ^a^	2.78 ± 0.2 ^b^	11.89 ± 0.35 ^a^	7.53 ± 0.65 ^b^
TPC [µg mL^−1^ of gallic acid]	2.45 ± 0.65 ^a^	0.82 ± 0.55 ^b^	4.85 ± 0.93 ^a^	2.31 ± 0.33 ^b^

Superscript letters (a and b) indicate significant differences between tested properties on pristine and deashed biochars (*p* < 0.05).

**Table 4:** Sorption and desorption magnitude of 2,4-D, MCPA, metolachlor, carbaryl and carbofuran on pristine (BC) and deashed biochar (BCd). Results are expressed as mean values ± standard deviation (*n* = 3).

Pesticide	Sorption		Desorption	
	BC	BCd	BC	BCd
2,4-D	7.3 ± 2.8 ^a^	94.4 ± 7.2 ^b^	77.8 ± 5.6 ^a^	13.2 ± 1.2 ^b^
MCPA	39.3 ± 8.0 ^a^	97.5 ± 3.0 ^b^	76.4 ± 5.1 ^a^	5.1 ± 0.2 ^b^
Metolachlor	93.4 ± 1.2 ^a^	96.4 ± 2.4 ^a^	1.5 ± 0.04 ^a^	0.5 ± 0.01 ^b^
Carbaryl	98.1 ± 0.1 ^a^	96.3 ± 0.1 ^b^	0.9 ± 0.2 ^a^	3.1 ± 0.1 ^b^
Carbofuran	88.0 ± 2.9 ^a^	92.7 ± 2.6 ^a^	5.4 ± 0.2 ^a^	5.9 ± 0.2 ^b^

Superscript letters (a and b) indicate significant differences between sorption and separately desorption magnitude of each agrochemical on pristine and deashed biochar (*p* < 0.05).

**Table 5:** Adsorption capacity (Q) of pristine (BC) and deashed biochar (BCd) towards the studied 2,4-D, MCPA, metolachlor, carbaryl and carbofuran pesticides. Results are expressed as mean values ± standard deviation (*n* = 3).

Q [mg g^−1^]	2,4-D	MCPA	Metolachlor	Carbaryl	Carbofuran
BC	0.16 ± 0.07 ^a^	0.82 ± 0.37 ^a^	1.96 ± 0.02 ^a^	3.35 ± 0.004 ^a^	1.75 ± 0.06 ^a^
BCd	2.32 ± 0.18 ^b^	2.03 ± 0.06 ^b^	2.02 ± 0.01 ^b^	3.30 ± 0.004 ^b^	1.84 ± 0.05 ^a^

Superscript letters (a and b) indicate significant differences between sorption and separately desorption magnitude of each agrochemical on pristine and deashed biochar (*p* < 0.05).

## Data Availability

Data from this study is available upon request.

## Supplementary Materials

The following are available online at https://www.mdpi.com/article/10.3390/ma16062185/s1. Table S1. Elemental composition of pristine (BC) and deashed (BCd) biochars determined with energy-dispersive X-ray spectroscopy (EDX); Figure S1. Calibration curve for the assessment of total phenolic content by Folin–Ciocâlteau method, expressed as gallic acid equivalent (mg of GA L^−1^ of extract).
