# Supplementary material for: Deashed Wheat-Straw Biochar as a Potential Superabsorbent for Pesticides
Source: Materials (Basel). 2023 Mar 9;16(6):2185. doi: 10.3390/ma16062185 (PMC10056329; doi:10.3390/ma16062185)
Supplement: Supplementary file 1 [file materials-16-02185-s001.zip › materials-2258930-supplementary.pdf]

Supplementary Materials:

Table S1. Elemental composition of pristine (BC) and deashed (BCd) biochars determined with energy-dispersive X-ray spectroscopy (EDX). The ± errors [%] are given in the brackets.

Element	BC			BCd		
	Weight [%]	Atomic [%]	C error [%]	Weight [%]	Atomic [%]	C error [%]
C	57.60	66.58	19.4	77.04	83.32	25.4
O	34.69	30.11	11.4	18.54	15.05	6.4
Si	3.64	1.80	0.2	0.21	0.10	0.0
Mg	0.32	0.18	0.0	0.35	0.19	0.0
S	0.04	0.02	0.0	0.16	0.06	0.0

*exp. Details: line K, Acc. Voltage: 20.0kV, Puls th.:2.47 kcps

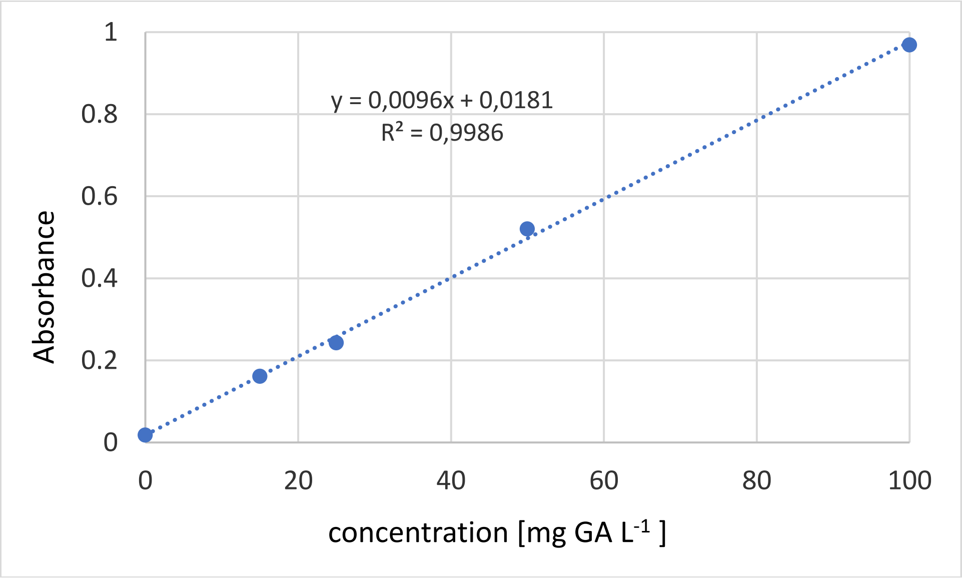

Figure S1. Calibration curve for the assessment of total phenolic content by Folin-Ciocalteau method, expressed as gallic acid equivalent [mg of GA L^{-1} of extract].
